# Long-term follow-up after stereotactic radiosurgery of intracanalicular acoustic neurinoma

**DOI:** 10.1186/s13014-017-0805-0

**Published:** 2017-04-21

**Authors:** Daniel Rueß, Lea Pöhlmann, Stefan Grau, Christina Hamisch, Alexandra Hellerbach, Harald Treuer, Martin Kocher, Maximilian I. Ruge

**Affiliations:** 1 Department of Stereotaxy and Functional Neurosurgery, Centre of Neurosurgery, University Hospital of Cologne, Kerpener Strasse 62, 50937 Köln, Germany; 2 Department of General Neurosurgery, Centre of Neurosurgery, University Hospital of Cologne, Kerpener Strasse 62, Cologne, 50937 Germany; 3 Department of Radiation Oncology, University Hospital of Cologne, Kerpener Strasse 62, 50937 Cologne, Germany

## Abstract

**Background:**

The management of solely intracanalicular acoustic neurinoma (iAN) includes observation, microsurgical resection and radiation therapy. Treatment goals are long-term tumor control, hearing preservation and concurrently low side-effects. Stereotactic radiosurgery (SRS) has evolved as an alternative first-line treatment for small AN. Here we report about the long-term follow-up of a unique cohort of patients with iAN after LINAC or Cyberknife® based SRS.

**Methods:**

In this single center retrospective analysis, we included all patients with iAN who underwent single session LINAC or Cyberknife® based SRS between 1993 and 2015, and who had a minimum follow-up period of six weeks. Patient data were analyzed in terms of radiological and clinical tumor control (no further treatment necessary), subjective preservation of serviceable hearing, objective change in pure tone averages (PTA), and adverse events rated by the Common Terminology Criteria for Adverse Events (CTCAE; v4.03).

**Results:**

Forty-nine patients (f/m = 21/28, median age 54 ± 12, range 20–77 years) were identified. Mean tumor volumes were 0.24 ± 0.12 cm^3^ (range, 0.1–0.68 cm^3^), the mean marginal dose was 12.6 ± 0.6 Gy (range, 11.0–14.0 Gy) and the prescription isodose was 75 ± 7.4% (range, 47–86%). Mean follow-up time was 65 months (range, 4–239 months).

Radiological tumor control was 100% during further follow-up. 17 (35%) out of 49 patients had lost serviceable hearing prior to SRS. Those with preserved serviceable hearing remained stable in 78% (*n* = 25/32) at the last follow-up (LFU). The median PTA (*n* = 16) increased from 25.6 dB prior to SRS to 43.8 dB at LFU.

Mild adverse events were observed temporarily in two patients (4%): one with CTCAE grade 1 facial nerve disorder after 3 months, resolving three months later, and one with CTCAE grade 2 facial muscle weakness resolving after 12 months. Three patients described permanent mild symptoms CTCAE grade 1 without limiting daily life (facial weakness *n* = 1, vertigo *n* = 2).

**Conclusion:**

SRS for iAN shows long-term reliable tumor control with a high rate of hearing preservation without considerable permanent side effects, and can be proposed as a safe and effective treatment alternative to microsurgical resection.

## Introduction

Acoustic neurinomas (AN) are primary intracranial tumors of the Schwann cell sheath surrounding the vestibulocochlear nerve (8th cranial nerve). The incidence of newly diagnosed AN has increased over the last 30 years from 3.1/1000000/y in 1976 up to 22.8/1000000/y in 2004 [1]. Through widespread availability of MR imaging, AN are diagnosed at earlier stages. Thus, the size of newly diagnosed AN decreased from 30 mm in 1979 to 10 mm in 2008 [1]. Concurrently, cases of exclusively intracanalicular AN (iAN) increased up to 25% [1], this fact represents a growing need for special counseling of these patients.

In the literature three management options are described: microsurgical removal, radiosurgery (or possibly fractionated radiation therapy) and “wait and scan “strategies (predominantly for patients at first diagnosis or with clinically and radiologically stable disease during follow-up) [2]. Desirable outcome goals are long-term tumor control, preservation of cranial nerve function, as well as functional hearing, and maintenance of a high quality of life.

Radiosurgery has evolved as an alternative first-line treatment for small and growing AN, and can achieve tumor control rates between 91 and 100% [2]. However, data about side effects, tumor control and outcome is scarce for the group of iAN treated with stereotactic radiosurgery (SRS).

To assess the long-term effectiveness of iAN management we performed a retrospective review of our 22-year database (1993–2015) of patients treated with LINAC or Cyberknife® based SRS.

## Methods

### Subjects and populations

In this single center retrospective analysis of a defined period (between 1993 and 2015) we included patients who were treated with single session stereotactic radiosurgery (SRS), either with modified linear accelerator (LINAC) or with Cyberknife® (CK) specifically for unilateral iAN. Further inclusion criteria requested a minimum of six weeks of clinical and radiological follow-up.

Documented baseline data included patient characteristics (age, gender, tumor volume, time span between duration of symptoms before diagnosis and treatment) and relevant radiosurgical parameters (coverage, prescribed dose, marginal dose). Indication of SRS treatment were.

Objective pre- and post-treatment hearing impairment was evaluated with available tone audiograms according to pure tone averages (PTA) as defined by the WHO [3]. According to the Gardner-Robertson-Scale [4], which includes PTA and a speech discrimination score, we additionally evaluated subjective pre- and post-treatment hearing. This involved asking the patients about their ability to use a phone with their affected ear. If communication using a phone was still possible without hearing aids we regarded this as functional (serviceable) hearing. In addition, if the PTA level was < 50 dB we regarded this as serviceable hearing referring to the Gardner-Robertson Grades I and II [4].

Further clinical evaluation was carried out by interviewing individual patients about tinnitus, vertigo, imbalance and facial motor and sensor function.

For the evaluation of side effects, we only collected reports of new symptoms after SRS. All symptoms were rated according to the Common Terminology Criteria for Adverse Events (CTCAE; v4.03, pp 51–55, chapter “Nervous system disorders”) [5]. We excluded the adverse event “acoustic nerve disorder” due to the fact that the patient already had impairments of the CN VIII as primary symptoms due to their iAN.

### Indication for SRS

We treated patients if iAN showed radiological tumor growth. Further, if there was a deterioration of objective deficits by means of measurable hearing loss or subjective worsening of symptoms e.g. vertigo, dizziness, tinnitus (group A). Otherwise treatment was offered when patient´s history revealed a worsening dynamic of subjective and/or objective symptoms or deficits leading to MRI diagnosis of iAN as course of these symptoms (group B). For further analysis, we divided our cohort following these indications in group A and B respectively. Patients with recurrent iAN were excluded from this analysis.

### Tumor control

For evaluation of radiological tumor control the pretreatment magnetic resonance imaging (MRI) data were compared to the last follow-up MRI images. Tumor size was defined as the maximum mediolateral (ml) and anterio-posterior (ap) diameter in transverse contrast-enhanced T1 MRI, since this was traditionally described in numerous retrospective studies [6]. An increase in tumor size of more than 3 mm was defined as tumor growth and therefore loss of local control according to Huang et al. [7]. Within the first 18 months after SRS, tumor enlargement accompanied by central hypointensity was defined as transient volume expansions as described before [8, 9]. According to earlier studies [10, 11] we additionally defined tumor control as freedom from planned or realized re-intervention (e.g. repeated radiosurgery or microsurgery). As described by Stangerup et al. [12] growth to extrameatal direction was also defined as growth and loss of tumor control. Clinical evaluations and MR images were normally performed at 6 months within the first year after radiosurgery, and annually thereafter in all patients.

### Radiosurgery with modified linear accelerator (LINAC)

For radiosurgical treatment with LINAC the patient’s head was immobilized under local anesthesia with a stereotactic frame (Riechert-Mundinger) followed by contrast enhanced and thin-slice high-resolution computed tomography (CT) for each patient. The CT and a high-resolution MRI (routinely after 1996) obtained before treatment were registered using the software STP (STP 3.3 and 3.5, Howmedica Leibinger, Freiburg, Germany). Subsequently, the tumor and its critical structures (e.g. brainstem, cerebellum, Trigeminal nerve) were outlined by a neurosurgeon experienced in stereotactic radiosurgery, and a treatment plan was generated by a medical physicist. The final irradiation plan was drawn up after interdisciplinary consensus between the stereotactic neurosurgeon, a radiation oncologist also experienced in SRS, and the medical physicist. Subsequently, the radiosurgical treatment was performed by using a linear accelerator (SL25, ELEKTA, 6 MEV photon beams) equipped with tertiary changeable collimators with 3–30 mm diameter openings and a non-coplanar rotational scheme (6–10 arcs ranging from 20°–160° to 200°–340°) as previously described by Ruge et al. [13].

### Radiosurgery with cyberknife®

Prior to Cyberknife® treatment a high-resolution contrast-enhanced CT was acquired and merged with a high-resolution contrast-enhanced MRI with T1- and T2-weighted images. Cyberknife® treatment planning was carried out with the software Multiplan v4.5. As with the planning for LINAC radiosurgery, the tumor and its critical structures were outlined by the same experienced team of SRS physicians and medical physicists.

For the Cyberknife® treatment the patient was comfortably immobilized on the Cyberknife® treatment table (Accuray, Sunnyvale, California) with a custom-made aquaplast mask. Usually Cyberknife® treatment was performed in an out-patient setting.

### Imaging techniques for SRS

Before 1996, the tumor was outlined at least on stereotactic CT images, although MR imaging was used for this purpose increasingly when available. The early version of the planning software did not allow the integration of MRI. Since 1996, the tumor was routinely outlined on MRI (Phillips, MR-Scanner 1.5 or 3 Tesla), which was obtained prior to SRS and integrated into stereotactic CT (1 mm slice thickness (st), Phillips 8-slice or 16-slice multidetector CT, since 2012 Toshiba 16-slice multidetector CT). Since the year 2008 we have used a standardized MRI protocol comprising a set of four MRI (3 Tesla) modalities: two T1-weighted contrast-enhanced sequences with 2 mm (T1 TFE 3D) and 1.2 mm (T1 FFE 3D) st, and two T2-weighted sequences with a st of 2 mm (T2 TSE) and 1 mm (T2 DRIVE 3D). Before 2008 we usually obtained only T1 TFE 3D and T2 TSE MRI (1.5 Tesla).

### Statistical analysis

Descriptive summaries were prepared for the patients’ demographics. The Wilcoxon-rank-sum test was used to compare clinical parameters between groups. A Kaplan Meier analysis was additionally used to evaluate hearing preservation, for example. The factors affecting serviceable hearing preservation were analyzed by a Cox proportional hazards model. The following variables were tested: age, gender, tumor volume (TV), transient volume expansion of tumor (TVE), time span between duration of symptoms prior to SRS, co-morbidities, radiation dose to the tumor margin, and the SRS system used. A *p* value of <0.05 was considered as statistically significant. The statistical analysis was performed using the software Graphpad PRZM 6.0 and SPSS 22.0.

## Results

### Patient collective

A total of 49 patients (f/m = 21/28) with a median age of 54 (range 20–77 years) were identified (Table 1). Median follow-up was 47 months (range, 4–239 months) and mean follow-up was 65 months ± 62.2 (SD). Besides the defined inclusion criteria of a minimum of six weeks post SRS we found the shortest observation period to be four months in two patients. Four patients (7.8%) of the collective had a recurrent iAN after surgery. The other 45 patients were treated either after detecting the diagnosis via MRI (group B, *n* = 24, 53%) or after a longer period of observation with MRI if there was tumor growth or clinical deterioration (group A, *n* = 21, 47%). In group A we found 86% (*n* = 18/21) progressive tumor growth and 76% (*n* = 16/21) worsening of symptoms before SRS. We found no significant difference between groups besides the time span between first MRI (diagnosis of iAN) and SRS (Table 1).

**Table 1:** Clinical characteristics and treatment of patients

Patient characteristics	
Total no. of patients	49
Gender (m:f)	28 : 21
Recurrent iAN	4 (8.2%)
Age (years)^a^	54 (20;77)
Tumor volume (cm^3^)^a^	0.24 (0.1;0.68)
Mean follow-up (months)^a^	65 (4;239)
Initial Symptoms and Signs	
Hearing loss	6 (12.2%)
Hearing disturbance (%)	40 (81.6%)
- Serviceable	29 (59.2%)
- Non-serviceable	11 (22.4%)
Vertigo (%)	17 (34.7%)
Imbalance (%)	10 (20.4%)
Tinnitus (%)	22 (44.9%)
CN VII impairment (%)	4 (8.2%)
Duration of symptoms	
Appearance of symptoms leading to SRS months)^a^	42.3 (4;182)
First MRI (diagnosis) to SRS (months)^a^	19.9 (2;153)
- Group A^a^	34 (9;134)
- Group B^a^	4 (2;6)
Radiation parameters	
LINAC (1993–2012)	38
CK (2013–2015)	11
Marginal dose (Gy)^a^	12.6 (11;14)
Dose prescription, isodose (%)^a^	75 (47;86)
Coverage (%) LINAC^a^	97.8 (91.1;100)
Coverage (%) CK^a^	98.7 (94.8;100)

If marked with ^a^data is given as mean. Range is given in brackets

The mean duration of symptoms leading to SRS in all patients was 42.3 months ± 40.7 (SD, range: 4–182). Between first MRI and radiosurgical treatment we found a mean time period of 19.9 months ± 28.9 (SD, range: 2–153).

LINAC SRS was performed between 1993 and 2012 in 38 patients. Since 2012, 11 patients were treated with robotic assisted SRS (Cyberknife®). The mean marginal dose delivered to all tumors independently of the radiation system was 12.6 ± 0.6 Gy (range, 11.0–14.0 Gy). The prescription isodose was 75 ± 7.4% (range, 47–86%).

### Tumor control

**Fig. 1:**
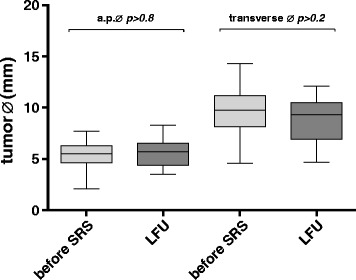
Comparison of tumor a.p. and transverse diameter (Ø). The tumor size does not differ significantly (*p* > 0.05) before and after SRS

After radiosurgery, tumors were monitored in all patients by follow-up MRI. At the last follow up (LFU) tumor size regressed in 10% (*n* = 5) and remained unchanged in 90% (*n* = 44) of cases. Morphological changes were seen in 61% (*n* = 30) of cases. Loss of central contrast was noted on follow-up imaging in 22 out of 49 (45%) patients. A TVE as previously described [9] followed by stabilization or regression was observed in 8 out of 49 (16%) patients. The average TV was 0.24 cm^3^ ± 0.1 (SD, range, 0.1–0.68) (Table 1). The average tumor size revealed an a.-p. diameter of 5.5 mm ± 1.2 (SD, range, 2.1–7.7) before SRS and 5.6 mm ± 1.3 (SD, range, 3.5–8.3) at LFU. The lateral dimension revealed before SRS was 9.5 mm ± 2.2 (SD, range, 4.6–14.3) and 8.9 mm ± 2.2 (SD, range, 4.7–12.1) at LFU. No significant difference was observed between the average tumor size before and after SRS at LFU (Fig. 1). Radiological tumor control (freedom from re-interventions) was 100% during further follow-up.

### Preservation of subjective hearing

**Fig. 2:**
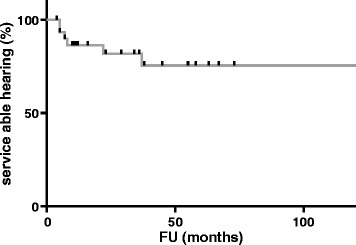
Kaplan Meier estimates a rate of hearing preservation with 86% after 12, 82% after 36, 75% after 60, and 56% after 120 months

Seventeen (35%) out of 49 patients had lost serviceable hearing prior to SRS, whereas 32 patients had subjective serviceable hearing prior to SRS. Those patients retained serviceable hearing in 78% (*n* = 25/32) of cases at LFU. The Kaplan-Meier estimates a preservation rate for subjective serviceable hearing of 75% after 120 months (Fig. 2). Four patients (8.2%) had improvements in their subjective hearing compared with preradiosurgery hearing. 36.7% (*n* = 18) of the collective reported subjective deterioration of hearing.

**Fig. 3:**
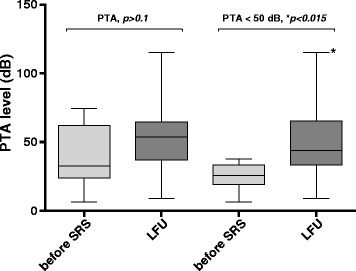
Comparison of PTA level before SRS and at LFU. In the patients with confirmed functional hearing (PTA < 50 dB, *n* = 10) median PTA levels increased significantly (*p < 0.015) before SRS and at LFU

In 16 patients additional PTA measurements were available and confirmed functional hearing < 50 dB in 10 patients. The median PTA of the whole collective increased from 32.5 dB prior to SRS to 53.8 dB at the last follow-up. The median PTA of patients with functional hearing increased significantly (*p* < 0.05) from 25.6 to 43.8 dB but remained under 50 dB (Fig. 3).

The following variables were tested in a multivariate analysis using the Cox proportional hazards model: age, gender, tumor volume, time span between duration of symptoms prior to SRS, co-morbidities, radiation dose to the tumor margin, and the radiation system used. We did not find any factors associated significantly (*p* < 0.05) with the odds of preserving serviceable hearing.

### Pre-, post-treatment symptoms and adverse events

**Fig. 4:**
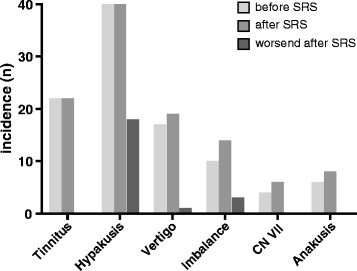
Incidence of symptoms given in absolute numbers before and after SRS. The third column depicts the deterioration of symptoms if this occurred

We compared the distribution of symptoms before and after SRS. 22 individuals reported tinnitus, 17 vertigo, 10 imbalance, eight had anakusis and four had a facial paralysis before SRS. After SRS we did not observe any new patient with tinnitus. Deterioration of symptoms was reported in the case of vertigo (*n* = 1) and imbalance (*n* = 2). An improvement in imbalance was reported by one individual. New transient or permanent symptoms after SRS were reported in nine cases (vertigo *n* = 2, imbalance *n* = 4, CN VII paralysis *n* = 2, facial hemispasm *n* = 1) (Fig. 4).

Adverse events were observed in five patients (10%). One patient developed a facial nerve disorder defined as facial hemispasm three months after SRS classified as CTCAE grade 1, which spontaneously resolved three months later. Another two patients developed facial muscle weakness classified as CTCAE grade 2 within the first six months, which resolved completely in one case and improved to CTCAE grade 1 in the other case one year after SRS. No impairments of trigeminal nerve function were reported in the collective. Another two patients had newly diagnosed dizziness, which we classified as CTCAE grade 1 since it did not limit activities of daily life.

In summary, we found three cases (6.1%) with new permanent symptoms after SRS classified as CTCAE grade 1.

## Discussion

Due to the increasing numbers of newly diagnosed iAN over the last four decades [1] counseling for affected patients has became more and more relevant. Nowadays three management options are recommended: surgery, “wait and scan” strategies and radiosurgery [2].

### Surgery

The surgical treatment of iAN can be performed using a great variety of approaches, e.g. translabyrinthine, middle cranial fossa, retrosigmoidal, transotic, retrolabyrinthine and transcochlear approaches [14]. So far, there is no general recommendation as to which approach should be preferred. The type of surgery still depends on the individual surgeon’s experience, especially when dealing with complications such as CN VII impairments [15, 16]. The most frequently recommended approaches for iAN surgery are translabyrinthine, media fossa and retrosigmoidal. Depending on the different approaches, microsurgical treatment of iAN implicates a varying risk for complications and prognosis. In the systematic literature review of Ansari et al. [17] comprising 35 studies with 5064 patients of which 428 had iAN, complete hearing loss after surgery was described for 40.3% up to 43.4% of cases. However, some series report better hearing preservation rates from 76.7 to 73.2% depending on the surgical approach [18]. Ansari et al report a risk for CN. VII disturbance in the subgroup of iAN ranging from 4 to 16% is associated with retrosigmoidal and middle fossa approaches [17]. The rate of tumor recurrence for all types of AN was described as 1.1% of patients treated with a middle crania fossa approach and 6.2% of those treated with a retrosigmoid approach [17].

In a meta-analysis of 32,680 patients with AN, Sughrue et al. described a mortality rate of 0.2% and a rate of neurological complications of 8.6% [19]. In case of small AN Sameshima et al. [18] report about a rate of surgical complications varying from 8.5% (retrosigmoidal approach) up to 11.6% (middle cranial fossa approach).

All of these aspects have to be taken into account when counseling the individual patient. In addition, not every patient is suitable for surgery due to co-morbidity, age, and individual preferences. In a comparison of current studies focusing on iAN (Table 2) we found tumor control rates from 97 to 100% while CN. VII impairment range from 0 to 2%. In our study we observe three cases of CN VII disturbances post SRS. Only one case led in a permanent impairment classified as CTCAE grade 1. Further, in our study we had no case of tumor recurrence. In summary, we see the risk for adverse events e.g. CN VII and tumor control rates is clearly in favor of radiosurgery.

**Table 2:** Characteristics of preexisting GK series and this series in the treatment of iAN

Authors, centre & year	n	Design	SRS System	FU (months)	Tumor control (no need for further treatment)	Preservation of hearing	CTCAE rating	Permanent CN VII impairment (%)
Pittsburgh, Ogunrinde et al. 1995 [22]	10	R, SC	GK	*mean:* 25 (3:64)	100%	80% (after one year)	no	0%
Pittsburgh, Niranjan et al. 1999 [23]	29	R, SC	GK	*median:* 33 (9;106)	100%	73%	no	0%
Pittsburgh, Niranjan et al. 2008 [10]	96	R, SC	GK	*mean:* 42 (12;144) *median:* 28	99%	64.5%	no	2%
Marseille, Regis et al. 2011 [11]	34	P, SC	GK	*mean:* 44 (9;222)	97%	64% (after 5 years)	no	n/a
Seoul, Kim et al. 2012 [9]	58	R, SC	GK	*mean:* 62 (24;111)	100%	55% (after 5 years)	no	n/a
Cologne, this series, 2016	49	R, SC	LINAC CK	*mean:* 65 (4;239) *median:* 47	100%	75% (after 5 years)	yes	2%

*P* prospective, *R* retrospective, *SC* single center

### “Wait and scan”

A widely discussed management option for iAN is the “wait and scan” strategy. Especially as a management option for patients with minimal symptoms, some authors recommend observation of iAN [2]. The small amount of tumor growth and the occurrence of spontaneous tumor regression over years seem to advocate a “wait and scan” strategy according to several observation studies [7, 12]. However, in contrast to these studies other authors describe 41% tumor growth to extrameatal extension at the first-year follow-up in the natural course of iAN [20]. Regis et al [11] found 74% tumor growth in their series after a median observation period of 33 months. Likewise, in our study we found a tumor growth rate of 86% after a median observation of 34 months in patients who were observed with MRI (group A). Following Bakkouri et al [20] the growth rates of intrameatal and extrameatal tumors did not differ significantly. In a meta-analysis of 34 studies comprising 982 patients Sughrue et al. reported a mean growth rate of 2.9 mm/year [12]. A higher hearing preservation rate existed in AN with a growth rate less than 2.5 mm/year. This supports the argument that most of the small tumors grow and need treatment at a proper time point.

In current studies focusing on iAN the rates of useful hearing preservation ranged from 41 to 74% within a 3.6–5-year observation period [11, 21]. However, Regis et al. [11] found in their prospective case-control study a rate of useful hearing preservation and concurrent tumor control with only 14% of cases after 5 years in the observation group compared to 60% in the cohort first-line treated with radiosurgery. Based on these results Regis et al. [11] support a pro-active treatment concept for iAN.

### Radiosurgery

Most of the series dealing with radiosurgery of AN combine the subgroup of iAN with medium and large tumors in their data analysis. Whereas our series describes a precise defined cohort of patients with iAN. So far only a few series address the radiosurgical treatment of solely iAN (Table 2). Furthermore, the only existing studies are Gamma-Knife (GK) series [9–11, 22, 23].

Our series is the first to report on the long-term results of a homogenous collective of patients with unilateral iAN treated with LINAC or CK. Thus, there is still a lack of comparable studies. Additionally, this unique series contains the longest mean and median follow-up duration after radiosurgery of iAN in literature. The tumor control rates showed similar result to the preexisting GK series. Furthermore, our study yields one of the longest rate of hearing preservations after 5 years. We can conclude that the different techniques of SRS do not differ significantly. Likewise, we found no significant difference in terms of tumor control, hearing preservation, or side-effects between LINAC and CK.

Additionally, in contrast to the other retrospective series (Table 2) we took the treatment strategy (indications for SRS) into account.

### Hearing preservation

There was no significant increase in median PTA before and after SRS in assessable patients (32%, *n* = 16). The median PTA increased significantly in patients with functional hearing (*n* = 10, 20%) but remained under the threshold value of 50 dB for loss of functional hearing [4] at the last follow-up. This is similar to preexisting iAN studies [9]. In our study the percentage of median PTA level increase was 60.4% before and after SRS. This correlates well with that of the natural course of iAN described by Pennings et al. [21], which was 73%. In fact, our value is slightly better and simultaneously we could achieve 100% tumor control whereas Pennings et al. [21] observed 40% of tumor growth after a median follow-up of 3.6 years. This supports the value of SRS in terms of hearing preservation and simultaneous tumor control as supported by other studies [10, 11].

In addition to the usual examination of hearing with PTA levels, we asked our patients about their subjective hearing. The rate of preserved subjective functional hearing is comparable to the other iAN studies (Table 2) with hearing preservation in three-quarters of patients after five years. One limitation of our study was the lack of a speech discrimination score and only a small amount of PTA examinations. Nevertheless, our study supports the favorable outcome in terms of hearing preservation in the case of radiosurgery.

### Side effects

In contrast to the other series we used the CTCAE criteria to rate post-treatment symptoms, complications or unexpected side effects of SRS. In our series we found a rate of 6.1% of permanent CTCAE grade 1. The already mentioned GK series of iAN either did not report on new symptoms [9, 11] or focused mainly on complications [10, 22, 23].

Niranjan et al. [10] observed new temporary facial paresis in 2% (*n* = 2) within the first four months after SRS. In our series we observe about 6% (*n* = 3) CN VII toxicity in which two cases only had temporary symptoms. In the literature a CN VII preservation rate is described as 100 – 94.9% when using radiation doses between 12 and 13 Gy [24]. We see our results as within this range and suggest that SRS may offer a reasonable treatment option for iAN with favorable treatment risk.

### Limitations of the study

The main limitation of the study is the retrospective character. This may lead to heterogenous patient collective, especially in terms of duration of symptoms before SRS. Due to the retrospective character, it is not possible to reconstruct exactly why some of the patients had such a long period of symptoms before first MRI was obtained. One explanation is that in the 90ties, early 2000 MRI were not so frequenty obtained than in nowadays. However, these heterogenous cohort presumably closely reflects current clinical practice.

Another weakness of the study is that FU of tumor size was only measured with maximum ml and ap diameter in transverse contrast-enhanced T1 MRI. One explanation is that a relevant amount of MRI is only available on paper print. Therefore, 3D volumetry is retrospectively only in scarce cases possible. We think that with our chosen method we make the cohort more comparable as it was also traditionally described in numerous retrospective studies [6].

In general, one can say that our study closely reflects the change of treatment prerequisites and improvements over time (regarding quality of imaging for contouring and follow-up).

## Conclusion

Exclusively iAN demands deliberate management. SRS shows reliable long-term tumor control with a high rate of hearing preservation. The rate of considerable permanent side effects is low. Thus, SRS can be proposed as a safe and effective treatment alternative to microsurgical resection or observation. Considering the strategy of observation, radiosurgical treatment should be taken into account especially if there is a deterioration of symptoms.

## Abbreviations

ap: Anterio-posterior
CK: Cyberknife®
CN: Cranial nerve
CT: Computed tomography
CTCAE: Common Terminology Criteria for Adverse Events
dB: Decibel
GK: Gamma-Knife
Gy: Gray
iAN: Intracanalicular acoustic neurinoma
LFU: Last follow up
LINAC: Linear accelerator
ml: Mediolateral
mm: Millimeter
MRI: Magnetic resonance imaging
PTA: Pure tone average
SD: Standard deviation
SRS: Stereotactic radiosurgery
st: Slice thickness
TV: Tumor volume
TVE: Transient volume expansion of tumor
y: Year
